# The Success of a Universal Hepatitis B Immunization Program as Part of Thailand’s EPI after 22 Years’ Implementation

**DOI:** 10.1371/journal.pone.0150499

**Published:** 2016-03-03

**Authors:** Nawarat Posuwan, Nasamon Wanlapakorn, Pattaratida Sa-nguanmoo, Rujipat Wasitthankasem, Preeyaporn Vichaiwattana, Sirapa Klinfueng, Viboonsak Vuthitanachot, Siriporn Sae-lao, Monthana Foonoi, Apinya Fakthongyoo, Jamorn Makaroon, Klaita Srisingh, Duangporn Asawarachun, Somchai Owatanapanich, Norra Wutthiratkowit, Kraisorn Tohtubtiang, Pornsak Yoocharoen, Sompong Vongpunsawad, Yong Poovorawan

**Affiliations:** 1 Center of Excellence in Clinical Virology, Department of Pediatrics, Faculty of Medicine, Chulalongkorn University, Pathumwan, Bangkok, Thailand; 2 Chumphae Hospital, Chumphae, Khon Kaen, Thailand; 3 Uttaradit Hospital, Mueang, Uttaradit, Thailand; 4 Lablae Hospital, Lablae, Uttaradit, Thailand; 5 Naresuan University Hospital, Mueang, Phitsanulok, Thailand; 6 Phra Nakhon Si Ayutthaya Hospital, Phra Nakhon Si Ayutthaya, Thailand; 7 King Narai Hospital, Mueang, Lop Buri, Thailand; 8 Narathiwat Ratchanakarin Hospital, Mueang, Narathiwat, Thailand; 9 Trang Hospital, Mueang, Trang, Thailand; 10 Bureau of Epidemiology, Department of Disease Control, Ministry of Public Health, Mueang, Nonthaburi, Thailand; Public Health England, UNITED KINGDOM

## Abstract

Hepatitis B vaccination for newborns was introduced in two provinces in 1988 as part of Thailand’s Expanded Program on Immunization (EPI), and extended to the whole country in 1992. Our previous studies showed that children and adolescents who were born after the implementation of this program had a carrier rate of less than 1%, compared with 5–6% before implementation. In 2014 we performed hepatitis B serosurveys among 5964 subjects in the different geographic regions of the country to evaluate the long-term immunogenicity and impact of universal hepatitis B vaccination in newborns as part of the 22-year EPI program, by assessing HBsAg, anti-HBc and anti-HBs seropositivity status. The number of HB virus (HBV) carriers, both children and young adults, who were born after universal HB vaccination was markedly reduced. The carrier rates among the age groups 6 months to 5 years, 5–10, 11–20, 21–30, 31–40, 41–50 and >50 years were respectively 0.1, 0.29, 0.69, 3.12, 3.78, 4.67 and 5.99%. The seropositivity rate for HBsAg in the post-EPI group was 0.6%, whereas in the pre-EPI group it was as high as 4.5% (*p*<0.001). HBV infection by means of detectable anti-HBc had also drastically declined in the population born after the HB vaccine was integrated into the EPI program. We estimated that the total number of HBV carriers amounted to 2.22 million, or 3.48% of the total population, most of whom are adults. The HB vaccine is the first vaccine shown to be effective in preventing the occurrence of chronic liver disease and hepatocellular carcinoma. Universal vaccination campaign will contribute to the eventual eradication of HBV-associated disease.

## Introduction

Hepatitis B virus (HBV) infection involves three-quarters of the world’s population, with chronic infection affecting approximately 350 million people worldwide [1]. It is highly endemic in South-East Asia, China, sub-Saharan Africa, and the Amazon Basin, with a rate of chronic infection of more than 8% among the general population in these areas. In areas where HBV infection is hyperendemic, the major route of transmission is perinatal or vertical, when an infected mother transmits the infection to her offspring [2]. Perinatal transmission can occur in utero, during delivery, or from close contact during the postpartum period. Early exposure to HBV, especially during infancy, contributes to as high as 70–90% of HBV chronic infection [3], and the risk increases when mothers are HBeAg positive or have a high viral load of HBV in the bloodstream [4,5]. Moreover, horizontal transmission of HBV is common in children living in sub-Saharan Africa [6]. HBV infection between 1 and 5 years of age poses a 25–50% risk of developing a chronic infection, whereas infected adults have a 1–5% chance of becoming a chronic carrier [1]. As a result, HBV infection is the major cause of chronic liver disease, cirrhosis, and hepatocellular carcinoma.

**Fig 1:**
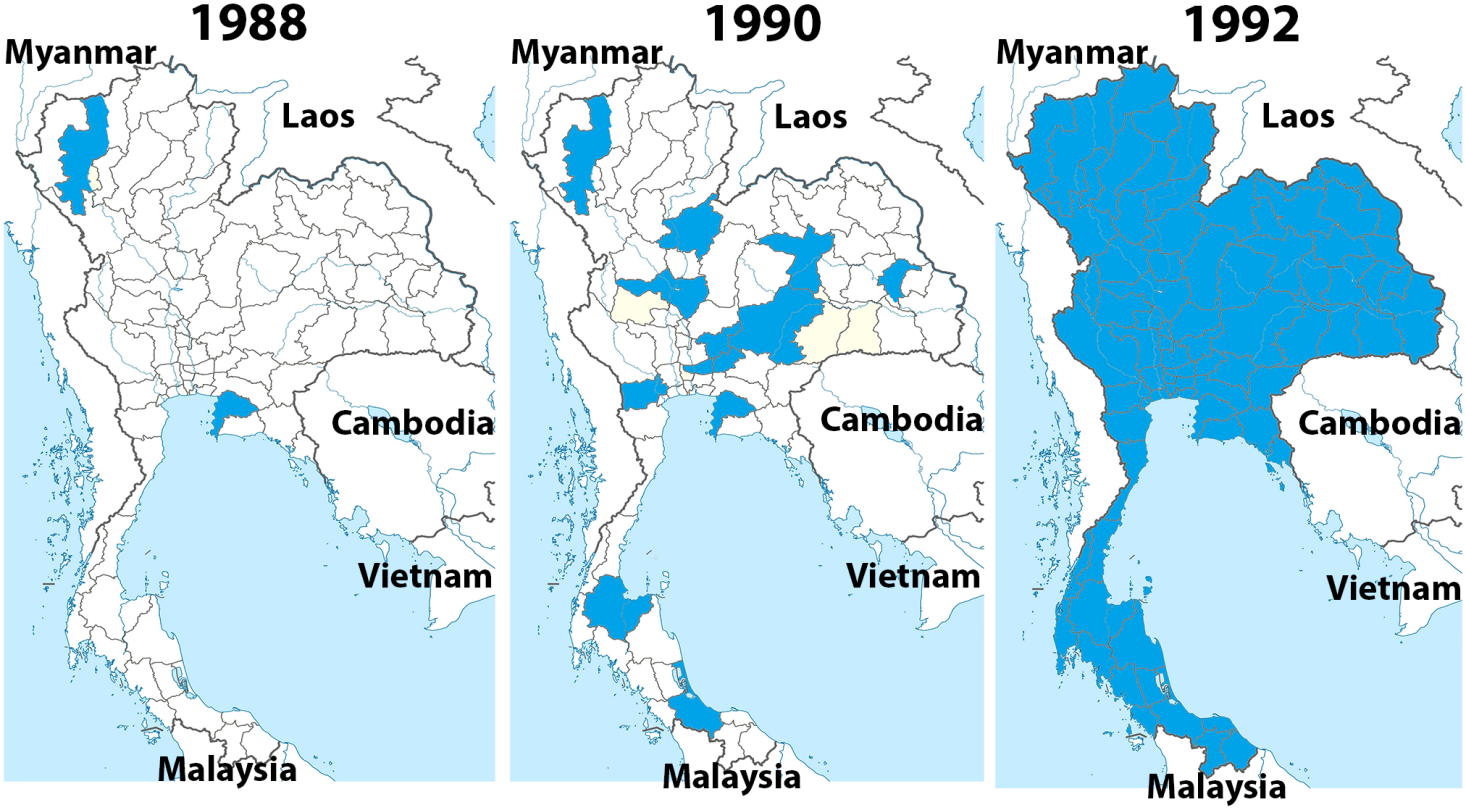
The implementation of universal HB immunization in 1988–1992. The Expanded Program on Immunization (EPI) began in two provinces, Chiang Mai and Chon Buri, in 1988, increased to 12 provinces in 1990, and covered the entire country by 1992.

In the past Thailand was an area highly endemic for HBV infection. A national policy led to the initiation of a pilot project to reduce HBV infection by incorporating the HB vaccine into the Expanded Program on Immunization (EPI), beginning in 1988 in the two provinces of Chiang Mai and Chon Buri. The initial vaccination schedule was three doses given at birth, 2 months, and 6 months of age. The second and third doses were given simultaneously with diphtheria, tetanus, and whole-cell pertussis (DTPw) vaccine, but at different injection sites. In 1990 the recommended schedule was expanded to 12 provinces, and universal HB vaccination for newborns was integrated into the EPI in 1992 (Fig 1) [7]. Universal hepatitis B vaccination for newborns was successfully implemented in Thailand before the World Health Organization (WHO) recommended that it should be incorporated into the EPI in 1992.

During this time the vaccine was given to Thai infants at 2 and 6 months of age simultaneously with the DTPw vaccine. The two injections not only caused more pain but also increased the cost of needles and syringes. Therefore, to increase compliance and coverage and reduce the cost of transportation and storage of the vaccine, the national policy introduced a combined DTPw-HB injection, but the HB vaccine schedule had to be changed to four doses. The first dose was given at birth and the second to fourth doses were given at 2, 4, and 6 months of age concomitantly with the DTPw vaccine. A study to evaluate the safety and immunogenicity of the combined DTPw-HB vaccine was conducted in Chiang Rai province over a 4-year period from 1994 to 1998 [8]. No differences were found in the safety or reactogenicity profiles, and the seroconversion rate for hepatitis B surface antibody was also high in infants who received the combined vaccine. Therefore, in 2005 the combined DTPw-HB vaccine was extended to 12 provinces, in 2006 to 24 provinces, and in 2007 to 27 provinces. In 2008 the program for the combined vaccine was implemented throughout the whole country.

With regards to immunization for infants born to HBsAg-positive mothers in Thailand, where HB immunoglobulin was not universally available, a study found that the risk of an infant becoming chronically infected was 3.74 times (95% CI = 0.97–14.39) higher if the interval between the first and the second doses exceeded 10 weeks [9]. This suggests that it is important for immunization programs to ensure timely second-dose vaccination to the infants of mothers with chronic HBV infection. Therefore, in 2009 the National Advisory Committee on Immunization in Thailand decided to recommend an extra dose of monovalent HB vaccine 1 month after the first dose in children born to such mothers.

After universal HB vaccination of infants had been implemented worldwide, there were consistent reports on its beneficial impact in reducing HBV infection rates in both highly and lowly endemic countries, including Thailand [10]. The carrier rate in children under 18 years decreased from 3.4% in the pre-vaccination era to 0.7% after universal vaccine implementation [7]. Moreover, long-term efficacy of the universal HB immunization showed persistently high anti-HBs levels (≥10 mIU/ml) in 64% of the population after 20 years of complete vaccination [11].

The aims of this study were to evaluate the impact of the universal hepatitis B vaccine in newborns (part of the EPI vaccination program since 1992) by assessing the seroprevalence of HBV markers in the different age groups of the Thai population. We evaluated the data in comparison to the 2004 report [12] and assessed the current burden of HBV in Thailand.

## Materials and Methods

The study protocol was approved by the Institutional Review Board of the Faculty of Medicine, Chulalongkorn University (IRB No.419/56), and the study was conducted in compliance with the principles of the Declaration of Helsinki under good clinical practice. Informed written consent was obtained from each participant or their parents, and all samples were treated as anonymous.

### Location of the study

**Fig 2:**
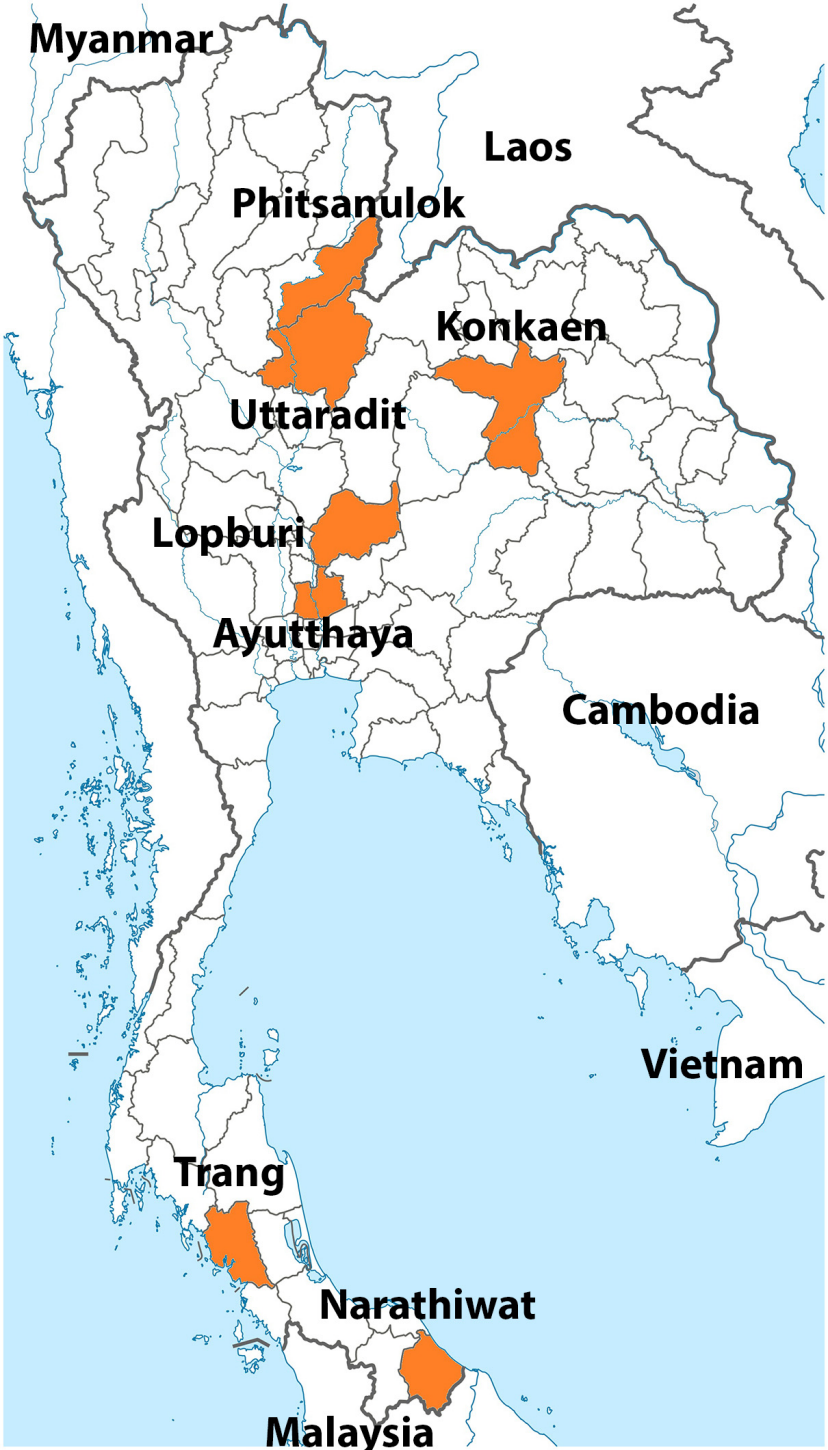
Map of Thailand showing the seven provinces in this study. Uttaradit and Phitsanulok represent the northern provinces; Lop Buri and Ayutthaya represent the central provinces; Trang and Narathiwat represent the southern provinces; and Khon Kaen represents a northeastern province (all denoted in orange).

Serum samples were collected from participants residing in seven provinces in four regions of Thailand (Fig 2). Uttaradit and Phitsanulok provinces represented the northern region of Thailand, Khon Kaen province represented the northeastern region, Ayutthaya and Lop Buri provinces represented the central region, and Narathiwat and Trang provinces represented the southern region.

### Population

The study was conducted between April and October 2014. A total of 5964 persons from the four regions were enrolled: 1411 from the northern region, 1637 from the northeastern region, 1535 from the central region and 1381 from the southern region. The inclusion criteria included age between 6 months and 60 years; no immunological disorders such as AIDS (acquired immunodeficiency syndrome), malignancy, or severe hematologic disorder; and no history of other chronic diseases. Those who were currently on immunosuppressants, steroids, immunosuppressive drugs or chemotherapy were excluded. We also excluded people who had congenital defects, chronic diseases, and any diseases that pose a risk for venipuncture, such as hemophilia and other blood disorders.

### Laboratory methods

Serum samples were analyzed at the Center of Excellence in Clinical Virology, Department of Pediatrics, Faculty of Medicine, Chulalongkorn University. All samples were treated as anonymous. HBV markers were detected by enzyme-linked immunoassay assay (ELISA) with automated Architect (Abbott, Wiesbaden, Germany), which included HBsAg, anti-HBs, and anti-HBc. The data were interpreted as positive using the cut-off point following the manufacturer’s instructions, which were >1 S/CO (sample to cut-off) for HBsAg and anti-HBc, and >1 mIU/ml for anti-HBs. Seroprotection was defined as >10 mIU/ml of anti-HBs.

### Data and statistical analysis

Data are presented in graphs and tables that showed the current seroprevalence of HBV markers in both numbers and percentages. The GMT (geometric mean titer) was calculated from anti-HBs titer >1 mIU/ml by multiplying individuals’ anti-HBs levels and taking the *n*th root of the product (where *n* is the number of observations). The GMT for each age group is presented on a graph with log_10_ scale. There were three provinces—Phitsanulok, Khon Kaen, and Ayutthaya—where HB vaccination for all newborns began in 1990, 2 years earlier than in the other four provinces. Therefore, participants residing in Phitsanulok, Khon Kaen, and Ayutthaya provinces were divided into two groups: those under 24 years of age were classified as ‘after EPI’ and those over 24 as ‘before EPI’. People residing in the other four provinces—Narathiwat, Trang, Uttradit, and Lop Buri—were classified as ‘before EPI’ if they were over 22 and as ‘after EPI’ if they were under 22. The results were analyzed by Microsoft Excel and a chi-squared contingency table to compare the seropositivity of HBV markers between those born before and after HB vaccination was integrated into the EPI program. The number of HBV carriers among the Thai population was calculated from the percentage of HBV carriers in each age group and the current Thai population data from official statistics registration systems, the Department of Provincial Administration, the Ministry of Interior of the Kingdom of Thailand. Data comparison was also made to the nationwide seroprevalence survey of HBV markers in 2004 in order to evaluate changes in the patterns of all HBV markers [12].

## Results

### Seroprevalence of HBsAg, anti-HBs, and anti-HBc before and after EPI

**Fig 3:**
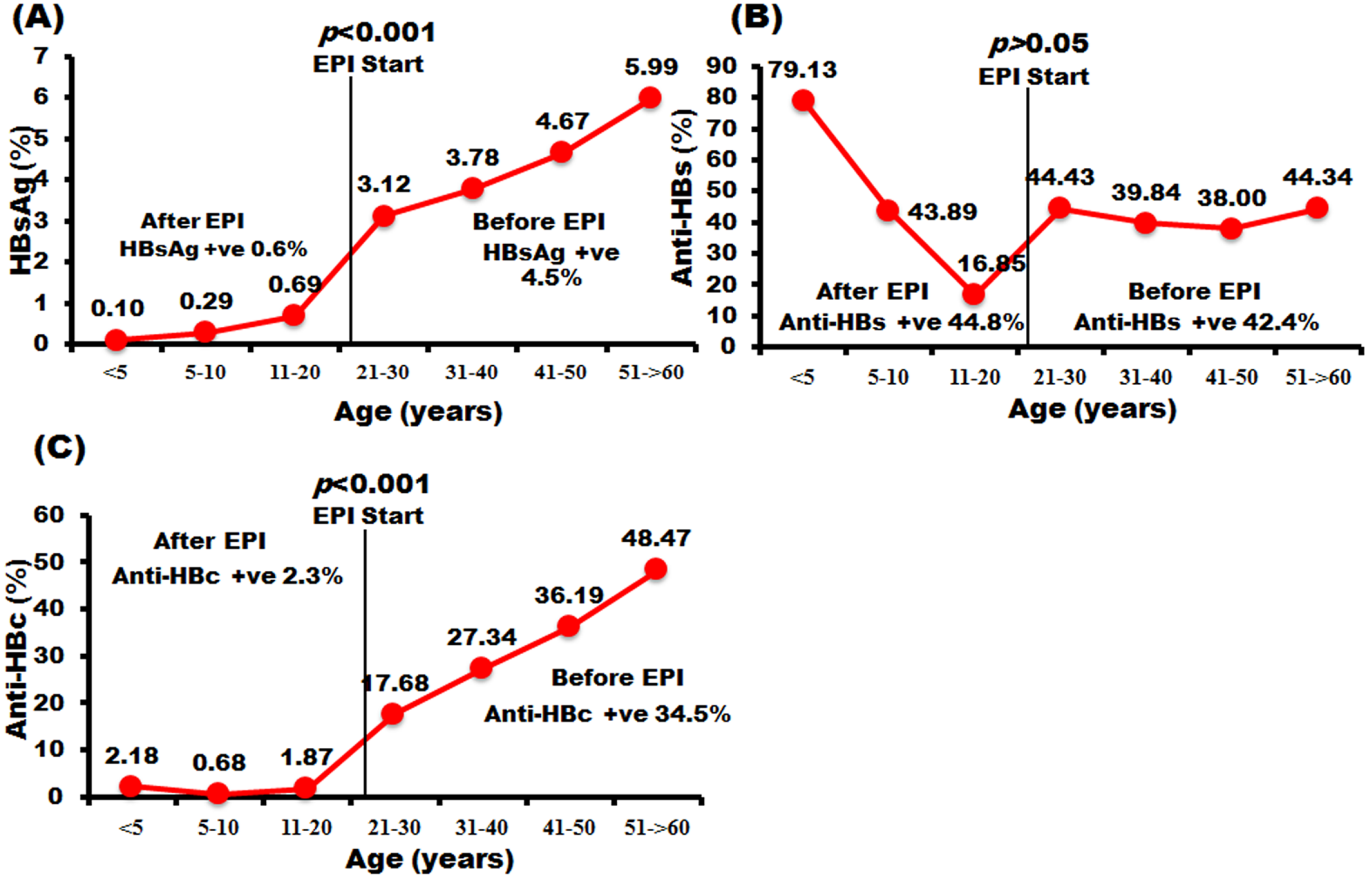
The prevalence of HBV assessed by seropositivity levels of HBsAg and anti-HBc and the seroprotective level of anti-HBs. Individuals were divided into seven age groups (<5, 5–10, 11–20, 21–30, 31–40, 41–50 and 51–60 years plus). The proportions of individuals positive for HBsAg (A), anti-HBs (B), and anti-HBc (C) are shown on the *y*-axis. The age at which EPI began is denoted by a vertical line. The ‘before EPI’ group was defined as individuals > 22–24 years of age (depending on the province). The ‘after EPI’ group was defined as individuals < 22–24 years of age.

We collected serum from 5964 participants aged 6 months to 60 years from seven provinces in four regions of Thailand. The seroprevalence of HBsAg, anti-HBs, and anti-HBc is shown in Fig 3. Participants under 5 years of age up to 22–24 years were classified as ‘after EPI’ and those over 22–24 years were classified as ‘before EPI’ (see Materials and Methods). The data showed that the prevalence of HBsAg increased with age. The seropositivity rate of HBsAg in the after-EPI group was 0.6%, whereas in the before-EPI group it was as high as 4.5% (*p*<0.001). The rate of seroprotectivity of anti-HBs was highest among infants under 5 years (79.1%), and declined gradually to 43.9% in children aged 5–10 and 16.9% in adolescents aged 11–20. However, among those born before EPI the seroprotectivity rate of anti-HBs was steady at approximately 38–44%. The average seroprotectivity rate of anti-HBs between the two EPI groups (before and after) was no different, at 42.4% and 44.8%, respectively (*p*>0.05). The patterns of anti-HBc seropositivity were similar to those of HBsAg, as the rate increased with age and there were statistically significant differences between the before and after groups: 34.5% and 2.3%, respectively (*p*<0.001). The seroprevalence of the three markers in each age group is described in more detail in S1 Table.

### Seroprevalence of HBsAg, anti-HBs, and anti-HBc among four regions of Thailand

**Fig 4:**
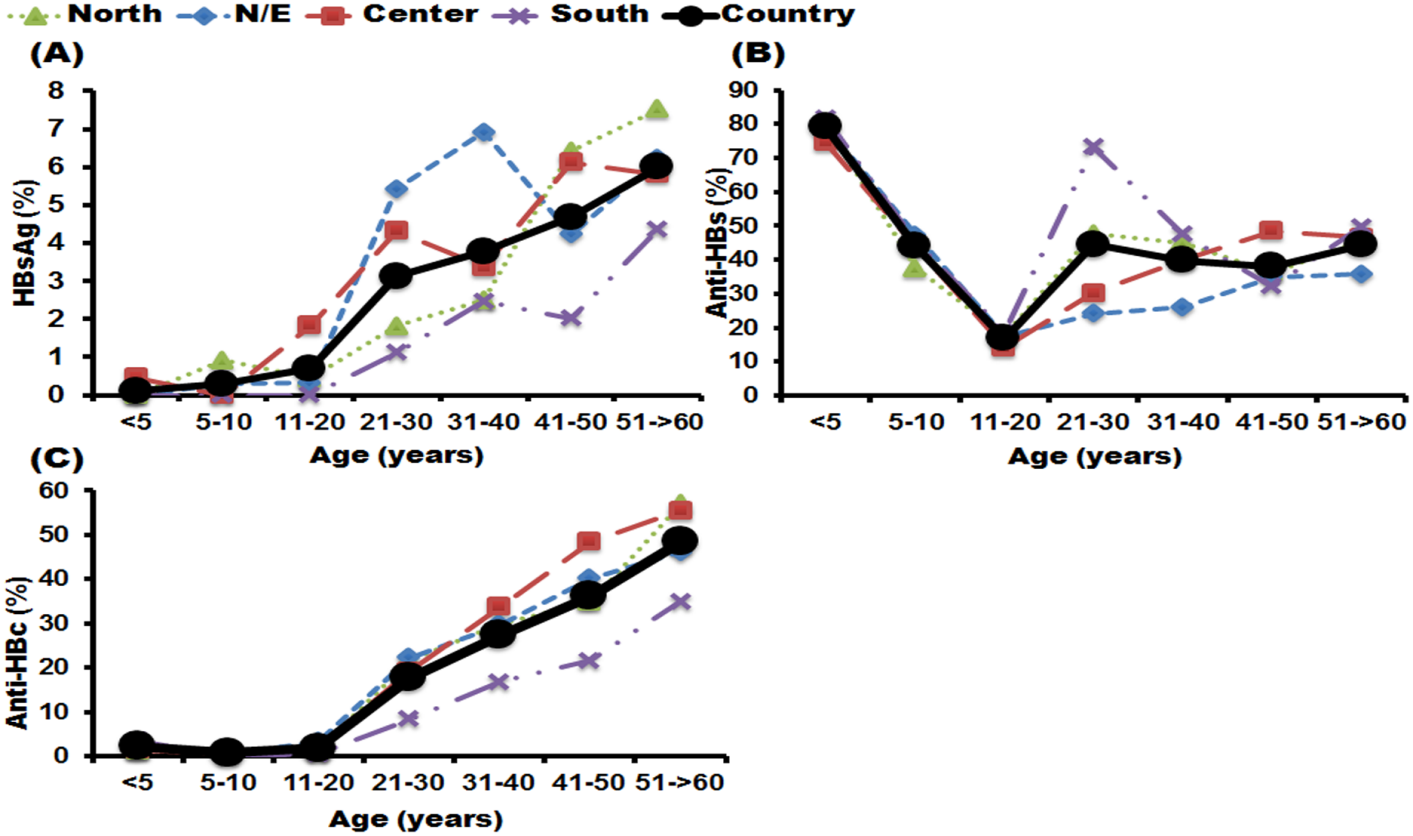
The prevalence of HBsAg, anti-HBs, and anti-HBc among populations residing in four parts of Thailand. The seven age groups were <5, 5–10, 11–20, 21–30, 31–40, 41–50 and 51–60 years plus (*x*-axis). The *y*-axis represents the percentage of the population with positive HBsAg (A), seroprotective anti-HBs (B) and positive anti-HBc (C).

The seroprevalence rates of HBsAg, anti-HBs, and anti-HBc among people residing in four regions of Thailand showed that less than 2% of people under 20 years of age in all regions were seropositive for HBsAg (Fig 4). However, among those over 20 the patterns were different between regions. In the population living in the northeastern part of Thailand, the highest seropositivity rate was found in the 31–40-year age group (6.91%), whereas the lowest rate was found in the 41–50-year group (4.23%). In contrast, among those living in the central region the highest seropositivity rate was found in 41–50-year-olds (6.12%, whereas 31–40-year-olds had the lowest rate, at 3.37%. People living in the south had the lowest overall rate of HBsAg positivity (1.38%). However, among people residing in the north the HBsAg seropositivity rate resembled the estimated data for the whole country. On the other hand, the seroprotectivity rate of anti-HBs in those ≤20 years old decreased with age in all populations, regardless of which region they lived in. Nevertheless, among people >20 years the anti-HBs seroprotectivity rates were slightly different. The 21–30-year group who lived in the southern region had the highest anti-HBs seroprotectivity rate (73.18%) compared to other regions. Finally, the seropositivity rate for anti-HBc in individuals age ≤20 years was similar in all four regions, with a range of 0.68–2.18%. In contrast, the trend was similar in all regions, as the seropositivity rate increased with age after 20 years of age. The seropositivity rate of anti-HBc was lowest in people living in the southern region.

### Geometric mean titers of anti-HBs

**Fig 5:**
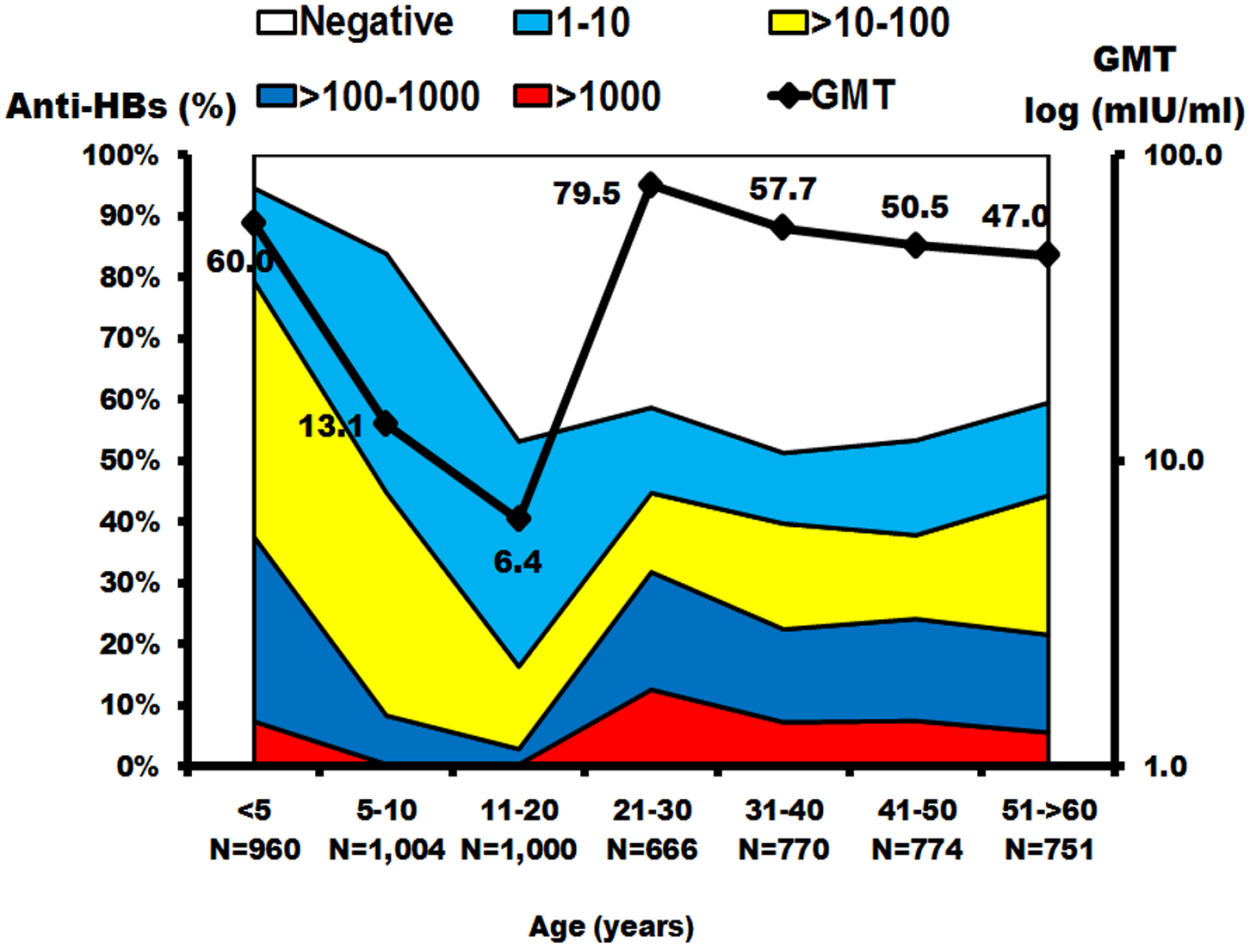
The prevalence of anti-HBs and geometric mean titers (GMTs) in the Thai population. The *x*-axis represents the seven age groups and the sample size in each age group. Scale on the left represents the percentage of the population with positive anti-HBs. Scale on the right represents the GMTs in each age group, with the means indicated as black squares. Antibody measurements were negative (white), 1–10 mIU/ml (blue), >10 100 mIU/ml (yellow), >100–1000 mIU/ml (dark blue) and > 1000 mIU/ml (red). GMTs were calculated from anti-HBs > 1 mIU/ml.

The geometric mean titer (GMT) was calculated from the anti-HBs concentration >1 mIU/ml, which indicated seropositivity. Examination of the anti-HBs titer and GMT in the Thai population across all ages showed that 79.4% of children <5 years had seroprotection from anti-HBs, and the GMT was 60 mIU/ml (Fig 5 and S2 Table). Among children 5–10 years of age the seroprotection rate decreased to 44.8% and GMT decreased to 13.1 mIU/ml. Meanwhile, adolescents aged 11–20 years had the lowest rate of seroprotection and GMT, at 16.4% and 6.4 mIU/ml, respectively. Nevertheless, the GMT of anti-HBs increased again in those over 20, ranging from 47.0 to 79.5 mIU/ml.

### Comparison of seroprevalence of hepatitis markers between 2004 and 2014

**Fig 6:**
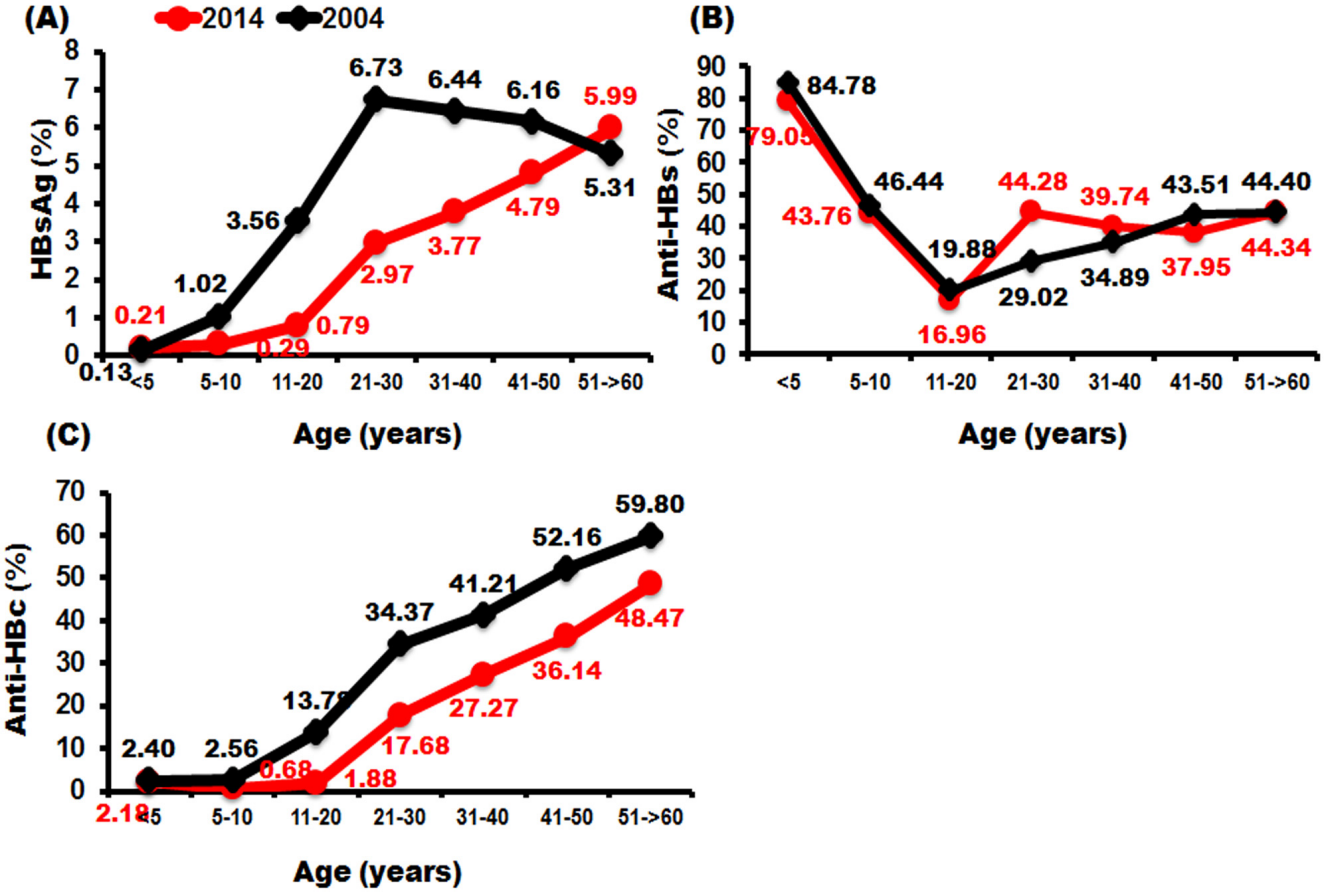
The percentage of (A) HBsAg seropositivity rate, (B) anti-HBs seroprotectivity rate and (C) anti-HBc seropositivity rate in the Thai population in 2004 and 2014. The red line represents the data in 2014, with the means indicated as red dots, and the black line represents the 2004 data, with means indicated as black dots.

We compared the HBsAg, anti-HBs, and anti-HBc seroprevalence rates between 2004 and 2014 in the Thai population (Fig 6). Overall, the HBsAg carrier rate declined from 1.4% to 0.4%, as did anti-HBc seropositive rates, from 5.5% in 2004 to 1.6% in 2014. Patterns of anti-HBc seropositive rates shifted to the right between 2004 and 2014, similar to the HBsAg carrier rate, except that in older people the shift in HBsAg carrier rate discontinued. On the other hand, the anti-HBs seroprotection rate had similar patterns between 2004 and 2014, except that in the 21–30-year age group there was a significant difference in the percentage of seroprotected individuals (44.28% vs. 29.02%, respectively).

### Estimation of numbers of HBV carriers among Thai population

**Fig 7:**
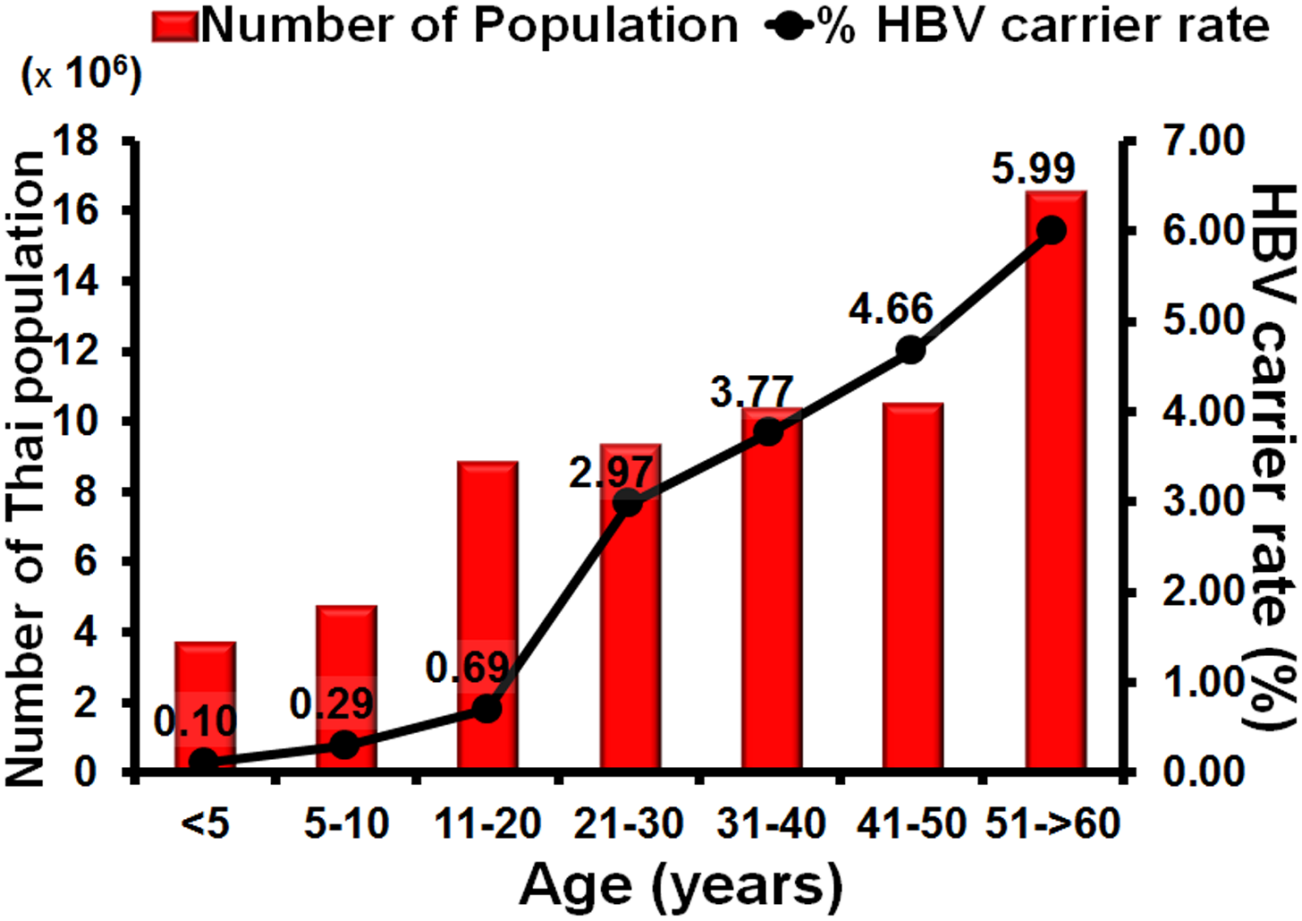
Estimation of the numbers of HBV carriers in the Thai population. The *x*-axis represents the seven age groups. Scale on the left represents Thai population. Scale on the right represents the percentage of HBV carriers, with the means indicated by black dots.

In this study the number of HBV carriers in Thailand was predicted using the percentage of HBsAg-positive samples in each age group and calculating back, based on the actual numbers of the Thai population in the official government records for each age group. In 2014 the Thai population consisted of 63,954,350 individuals and approximately 2,222,540 HBV carriers were found, including 3875 children aged under 5 years, 13,889 children aged 5–10 years, and 61,313 adolescents aged 11–20 years. There were 277,289, 389 671, 488,043, and 988,459 HBV carriers aged 21–30, 31–40, 41–50, and 51–60 and over, respectively. Based on the calculation of carriers in each age group, approximately 3.48% of the Thai population are carriers for HBsAg (Table 1 and Fig 7).

**Table 1:** The approximate number of HBV carriers by age in the Thai population.

Age	Number compared with the actual population		
(yrs)	Population	% HBV carrier rate	Number of HBV carriers^§^
<5	3,735,837	0.10	3,875
5–10	4,750,137	0.29	13,889
11–20	8,829,060	0.69	61,313
21–30	9,330,783	2.97	277,289
31–40	10,346,437	3.77	389,671
41–50	10,465,811	4.66	488,043
51– >60	16,496,285	5.99	988,459
Total	**63,954,350**	3.48*	**2,222,540**

^§^ Calculated from % HBV carrier rate in each age group multiplied by the population for each age group.

* Defined as the % HBV carrier rate calculated from the total number of HBV carriers (2 222 540) compared to the total Thai population of 63 954 350.

## Discussion

**Fig 8:**
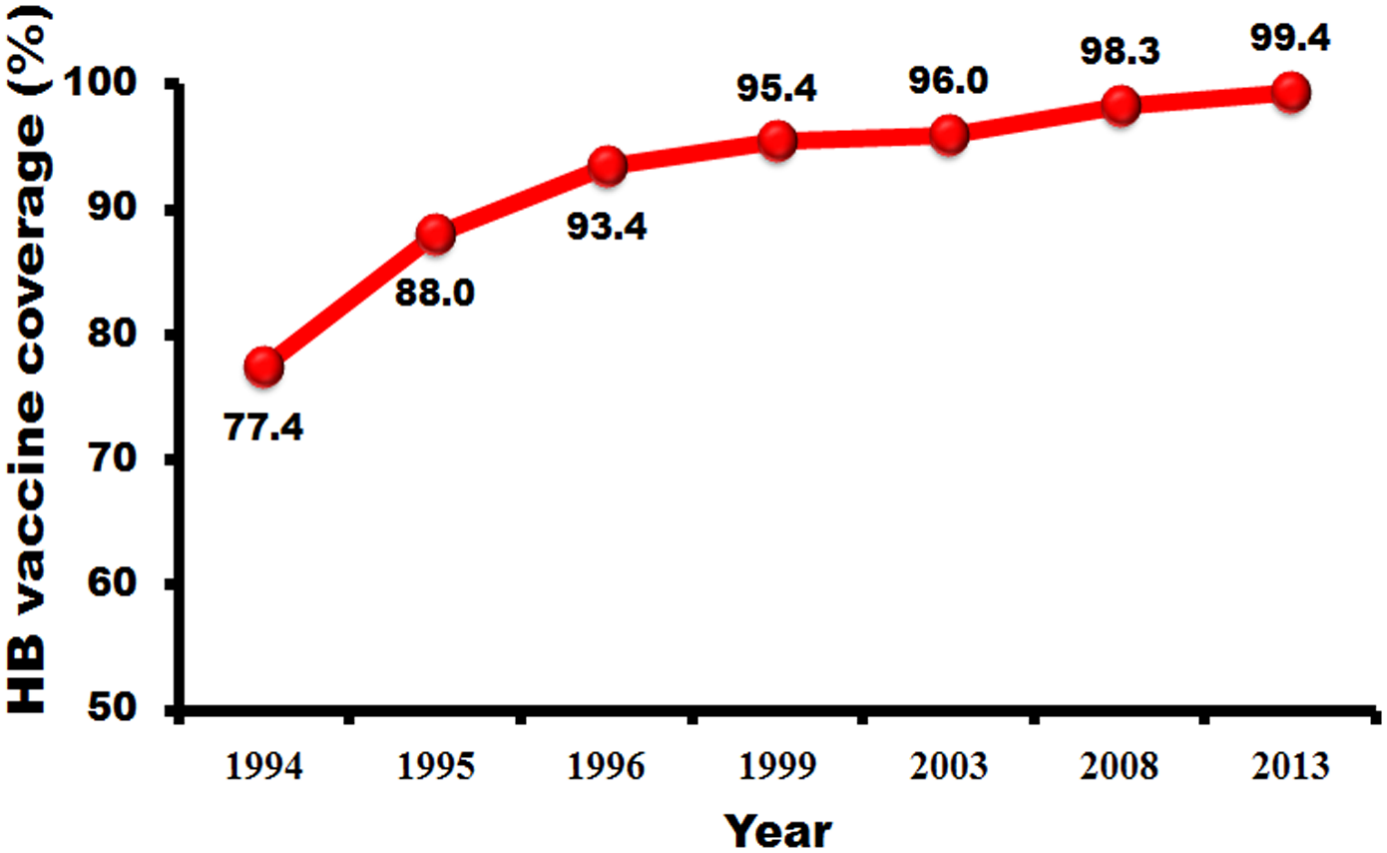
The percentage of hepatitis B vaccine coverage after the vaccine was integrated into the EPI program.

The universal hepatitis B vaccination, which has been integrated into the Thai EPI program since 1992, has resulted in a diminished seropositivity rate for both HBsAg and anti-HBc, the two markers that indicate carrier rate and natural infection for HBV in Thailand. A previous report which screened new blood donors for HBsAg [13] stated that in 1988 the seroprevalence of HBsAg was 7.1% during the pre-vaccination era. Our study has shown that in 2014 the prevalence had decreased to 2.38% when calculated on the sample population, or 3.48% when calculated from the percentage of seropositive samples in each age group and the actual number of the Thai population. The decrease was prominent among the younger generation, who received the HB vaccine fairly recently. According to our study, only 0.6% of Thai individuals who were born after the EPI was implemented were HBsAg positive. Moreover, since 1999 the coverage of HBV vaccination in Thailand has reached more than 95% (Fig 8), which indicates that in the future, when HBV program has expanded further, more and more people will have immunity to HBV and be less likely to contract the disease and transmit it to other people. The seropositivity to anti-HBc also decreased dramatically in people born after EPI. These results confirm that the rate of natural infection has declined in the post-vaccination era.

Several studies on the hepatitis serosurvey in Thailand reported that universal HBV vaccination, starting in 1992, resulted in a continuous decline in HBsAg carrier and HBV natural infection rates. In 1999, Poovorawan et al. [7] conducted a serosurvey to evaluate the impact of the HBV vaccine by analyzing the countrywide prevalence of HBV infection and the carrier rate in children aged 6 months to 18 years. The results showed that in children who were born after EPI, the seroprevalence of HBsAg was 0.7%, which was lower than in those who were born before EPI (3.4%). The anti-HBc seropositivity rate in children who were born before EPI was 15.7%, whereas in children born after EPI it was 6.3%. Five years later, in 2004, Chongsrisawat et al. investigated the seropositivity rate of three HB markers, again in a broader range of population from 6 months to 60 years of age [12]. Overall, the HBsAg, anti-HBs, and anti-HBc seropositivity rates amounted to 4%, 41.6%, and 26.5%, respectively. The findings of our study show that the overall HBsAg, anti-HBs, and anti-HBc seropositivity rates were 2.38%, 43.81%, and 17.09%, respectively. The seropositivity rate of HBsAg and anti-HBc decreased, whereas that of anti-HBs was similar to the reported in 2004. The decrease in the HBsAg rate was prominent in those aged 11–40 years as a result of 22 years’ universal vaccination, but not among people older than 40, as older people may have already acquired HBV and remained seopositive for a long period. The decline in seropositivity of anti-HBc was similar in all age groups, with a pattern of right-shift to an interval of 10 years as universal vaccination reduced the rate of natural infection in all age groups. However, the pattern of anti-HBs seropositivity rate in the 2004 and 2014 serosurveys was similar, except that the 21–30-year age group in the 2014 study had a higher seroprotection rate than in 2004 (44.2% vs. 29.2%). In recent years the national policy has recommended catch-up vaccination for adolescents and young adults who missed the HB vaccination at birth, in order to achieve three doses of vaccine. This could result in higher HBs titers.

The seroprevalence rates of all three markers were slightly different between the four regions of Thailand. In those living in the northeastern part, the highest HBsAg seropositivity rate was found in the 31–40-year group (6.91%), whereas in those living in the central region the highest HBsAg seropositivity rate was found in 51–60-year-olds and over (7.53%). People in the south had the lowest HBsAg seropositivity rate. The slight differences in the peak age groups for HBsAg seroprevalence were probably due to the small sample size of adults in this 2014 survey, which could result in broader variations in seropositivity rates.

An earlier report [14] investigating ‘a’ determinant mutations in children born to HBV-carrier mothers found that the prevalence of the ‘a’ determinant was not significantly different between children in vaccinated and those in non-vaccinated groups. A recent study in 2012 evaluated 14 ‘complete vaccinees’ who were born to HBsAg-positive mothers and who contracted HBV. Around 14% had ‘a’ determinant mutations. However, these findings had some limitations owing to the small number of samples [15]. So far, the HB vaccine has been proved to be effective, as there has been no ‘vaccine escape mutation’, particularly the ‘a’ determinant, detected among the Thai population after 22 years of implementation, according to the study conducted by Yimnoi et al. [16].

When the population was divided into two groups by the age at which the vaccine was implemented, the results showed that the seroprotectivity rates of anti-HBs in the ‘before’ and ‘after’ EPI groups were different. Among those born after EPI, anti-HBs seroprotectivity declined according to age and reached its lowest level at 11–20 years. Several studies have shown that protective levels of anti-HBs persisted in around 20–25% of vaccinees after the long-term follow-up to 18–20 years, but none has evidence of HBV infection by means of negative detectable antiHBc [17]. Wang et al. also provided evidence in support of the waning vaccine-induced immunity when they reported a long-term study on the immunogenicity of the HBV vaccine given to infants, showing the waning of anti-HBs to <10 mIU/ml in the second decade of life, and less than one-quarter of the ‘complete’ vaccinees had a seroprotective level of anti-HBs [18]. The booster effect or anamnestic response occurred rapidly, with an acceptable level of detectable protective antibody (anti-HBs) in >90% of the participants after a challenge with either pediatric or adult doses of HB vaccine [19, 20–24]. HBV infection has a long incubation period (1–6 months), and the secondary response to the antigen is sufficient and early enough to protect against the infection. Poovorawan et al. studied the long-term efficacy of recombinant hepatitis B vaccine in infants in hyperendemic countries such as Thailand, who were born to carrier mothers and in whom the first dose was given at birth and the third and fourth doses were completed within 1 year [20]. The participants were followed for up to 20 years, and the results showed that after the age of 1 year no child was a carrier. Some of them experienced a natural booster effect due to exposure to HB, as detected by the rise in anti-HBs antibody during the follow-up period. In a very few cases asymptomatic HBV infection can occur by means of transient detectable anti-HBc. Routine screening for HBV antibody and the booster HB vaccine are not necessary in HBV hyperendemic countries. However, for individual care in high-risk groups (e.g. men who have sex with men (MSM), intravenous drug users (IVDU), immunosuppressed patients, those with an HBV-carrier spouse), HBV screening and a booster challenge are optional preventive efforts [25].

The results from this study, which yielded 2.38% seroprevalence derived from surveying approximately 6,000 individuals, was used to calculate the number of HBV carriers in the Thai population. The percentage of HBsAg seropositivity for each age group was extrapolated and suggested a HBV carrier rate of approximately 3.48% nationally. This represents approximately 2.2 million people who are HBsAg carriers, nearly half of whom are individual over 51 years of age and amount to almost 1 million people. This is a sequela from the pre-vaccination era, and medical personnel and resources are currently focused mainly on screening for hepatocellular carcinoma and treatment of HBV disease in this group. In the future, when vaccinated children grow up to become adults, this burden will decrease.

Several studies have shown that immunization with the HB vaccine prevents the development of persistent carriers, and reduces the incidence of acute hepatitis and hepatocellular carcinoma [4, 26, 27]. Unfortunately, disease surveillance in Thailand did not include the annual incidence of hepatocellular carcinoma and acute hepatitis B infection. As a result, it was hard to demonstrate the decrease in the incidence of those diseases after the universal vaccination program in Thailand. In addition, the sample population was not directly proportionate to the population density of Thailand in each age group. As a result, there could be some discrepancy between the estimate of actual numbers of carriers and seroprevalence status.

This study has shown the declining rates of HBsAg carriers and natural HBV infection in people who were born after the universal HB vaccine was included in the EPI program. As a result, we can expect a reduction in the rate of HBV-associated diseases such as chronic hepatitis, cirrhosis, and hepatocellular carcinoma. The HBV vaccine has been successfully implemented in many countries for more than 20 years, with good coverage and efficacy. A continuous serosurvey of HBV markers should be instituted in order to demonstrate the population-based persistence of anti-HBs as well as the status of HBV carriers.

## Supporting Information

S1 Table The prevalence of HBsAg, anti-HBs, and anti-HBc between four provinces in Thailand: northern, northeastern, central, and southern. (DOC)

S2 Table Anti-HBs titer and GMT in the study Thai population according to age. (DOC)
