# Supplementary material for: The Success of a Universal Hepatitis B Immunization Program as Part of Thailand’s EPI after 22 Years’ Implementation
Source: PLoS One. 2016 Mar 3;11(3):e0150499. doi: 10.1371/journal.pone.0150499 (PMC4777547; doi:10.1371/journal.pone.0150499)
Supplement: S1 Table — (DOC) [file pone.0150499.s001.doc]

**S1 Table. The prevalence of HBsAg, anti-HBs, and anti-HBc between four provinces in Thailand: northern, northeastern, central, and southern.**

| **Age** | **Number of participants** | | | | | **HBsAg** | | | | | **Anti-HBs** | | | | | **Anti-HBc** | | | | |
| --- | --- | --- | --- | --- | --- | --- | --- | --- | --- | --- | --- | --- | --- | --- | --- | --- | --- | --- | --- | --- |
| **(yrs)** | **N/E** | **Center** | **North** | **South** | **All** | **N/E** | **Center** | **North** | **South** | **All** | **N/E** | **Center** | **North** | **South** | **All** | **N/E** | **Center** | **North** | **South** | **All** |
| <5 | 251 | 217 | 238 | 257 | 963 | 0 | 1 (0.46) | 0 | 0 | 1 (0.10) | 199 (79.28) | 162 (74.65) | 191 (80.25) | 210 (81.71) | 762 (79.13) | 6 (2.39) | 3 (1.38) | 3 (1.26) | 9 (3.50) | 21 (2.18) |
| 5-10 | 339 | 320 | 218 | 146 | 1023 | 1 (0.29) | 0 | 2 (0.92) | 0 | 3 (0.29) | 160 (47.20) | 141 (44.06) | 82 (37.61) | 66 (45.21) | 449 (43.89) | 2 (0.59) | 2 (0.63) | 3 (1.38) | 0 | 7 (0.68) |
| 11-20 | 307 | 273 | 226 | 209 | 1015 | 1 (0.33) | 5 (1.83) | 1 (0.44) | 0 | 7 (0.69) | 53 (17.26) | 39 (14.29) | 40 (17.70) | 39 (18.66) | 171 (16.85) | 10 (3.26) | 5 (1.83) | 3 (1.33) | 1 (0.48) | 19 (1.87) |
| 21-30 | 166 | 162 | 166 | 179 | 673 | 9 (5.42) | 7 (4.32) | 3 (1.81) | 2 (1.12) | 21 (3.12) | 40 (24.10) | 49 (30.25) | 79 (47.59) | 131 (73.18) | 299 (44.43) | 37 (22.29) | 31 (19.14) | 36 (21.69) | 15 (8.38) | 119 (17.68) |
| 31-40 | 188 | 178 | 200 | 202 | 768 | 13 (6.91) | 6 (3.37) | 5 (2.50) | 5 (2.48) | 29 (3.78) | 49 (26.06) | 71 (39.89) | 90 (45.00) | 96 (47.52) | 306 (39.84) | 56 (29.79) | 60 (33.71) | 60 (30.00) | 34 (16.83) | 210 (27.34) |
| 41-50 | 189 | 196 | 187 | 199 | 771 | 8 (4.23) | 12 (6.12) | 12 (6.42) | 4 (2.01) | 36 (4.67) | 66 (34.92) | 95 (48.47) | 68 (36.36) | 64 (32.16) | 293 (38.00) | 76 (40.21) | 95 (48.47) | 65 (34.76) | 43 (21.61) | 279 (36.19) |
| 51->60 | 193 | 189 | 186 | 183 | 751 | 12 (6.22) | 11 (5.82) | 14 (7.53) | 8 (4.37) | 45 (5.99) | 69 (35.75) | 88 (46.56) | 85 (45.70) | 91 (49.73) | 333 (44.34) | 89 (46.11) | 105 (55.56) | 106 (56.99) | 64 (34.97) | 364 (48.47) |
| Total | 1633 | 1535 | 1421 | 1375 | 5964 | 44 (2.69) | 42 (2.74) | 37 (2.60) | 19 (1.38) | 142 (2.38) | 636 (38.95) | 645 (42.02) | 635 (44.69) | 697 (50.69) | 2613 (43.81) | 276 (16.90) | 301 (19.61) | 276 (19.42) | 166 (12.07) | 1019 (17.09) |
