# Supplementary material for: The Success of a Universal Hepatitis B Immunization Program as Part of Thailand’s EPI after 22 Years’ Implementation
Source: PLoS One. 2016 Mar 3;11(3):e0150499. doi: 10.1371/journal.pone.0150499 (PMC4777547; doi:10.1371/journal.pone.0150499)
Supplement: S2 Table — (DOC) [file pone.0150499.s002.doc]

**S2 Table. Anti-HBs titer and GMT in the study Thai population according to age.**

| **Age** | **<5** | **5-10** | **11-20** | **21-30** | **31-40** | **41-50** | **51->60** |
| --- | --- | --- | --- | --- | --- | --- | --- |
| **Participants** |  |  |  |  |  |  |  |
| **(yrs)** | **n=960** | **n=1004** | **n=1000** | **n=666** | **n=770** | **n=774** | **n=751** |
| **Anti-HBs titer (mIU/ml)** |  |  |  |  |  |  |  |
| Negative | 5.4 | 16.1 | 46.8 | 41.3 | 48.7 | 46.6 | 40.5 |
| 1–10 | 15.2 | 39.0 | 36.8 | 14.0 | 11.6 | 15.5 | 15.2 |
| >10–100 | 41.8 | 36.5 | 13.5 | 12.9 | 17.3 | 13.7 | 22.8 |
| >100–1000 | 30.2 | 8.0 | 2.5 | 19.2 | 15.2 | 16.7 | 16.0 |
| >1000 | 7.4 | 0.4 | 0.4 | 12.6 | 7.3 | 7.5 | 5.6 |
| **GMT (log mIU/ml)** |  |  |  |  |  |  |  |
|  | 60.0 | 13.1 | 6.4 | 79.5 | 57.7 | 50.5 | 47.0 |
